# Fluid Jet Stimulation of Auditory Hair Bundles Reveal Spatial Non-uniformities and Two Viscoelastic-Like Mechanisms

**DOI:** 10.3389/fcell.2021.725101

**Published:** 2021-08-26

**Authors:** Anthony W. Peng, Alexandra L. Scharr, Giusy A. Caprara, Dailey Nettles, Charles R. Steele, Anthony J. Ricci

**Affiliations:** ^1^Department of Physiology and Biophysics, University of Colorado Anschutz Medical Campus, Aurora, CO, United States; ^2^Department of Otolaryngology, Head and Neck Surgery, School of Medicine, Stanford University, Stanford, CA, United States; ^3^Neuroscience Graduate Program, School of Medicine, Stanford University, Stanford, CA, United States; ^4^Neuroscience Graduate Program, University of Colorado Anschutz Medical Campus, Aurora, CO, United States; ^5^Department of Mechanical Engineering and Aeronautics and Astronautics, School of Engineering, Stanford University, Stanford, CA, United States; ^6^Department of Molecular and Cellular Physiology, School of Medicine, Stanford University, Stanford, CA, United States

**Keywords:** stereocilia bundle, fluid jet, hair cell, modeling, creep, viscoelasticity

## Abstract

Hair cell mechanosensitivity resides in the sensory hair bundle, an apical protrusion of actin-filled stereocilia arranged in a staircase pattern. Hair bundle deflection activates mechano-electric transduction (MET) ion channels located near the tops of the shorter rows of stereocilia. The elicited macroscopic current is shaped by the hair bundle motion so that the mode of stimulation greatly influences the cell’s output. We present data quantifying the displacement of the whole outer hair cell bundle using high-speed imaging when stimulated with a fluid jet. We find a spatially non-uniform stimulation that results in splaying, where the hair bundle expands apart. Based on modeling, the splaying is predominantly due to fluid dynamics with a small contribution from hair bundle architecture. Additionally, in response to stimulation, the hair bundle exhibited a rapid motion followed by a slower motion in the same direction (creep) that is described by a double exponential process. The creep is consistent with originating from a linear passive system that can be modeled using two viscoelastic processes. These viscoelastic mechanisms are integral to describing the mechanics of the mammalian hair bundle.

## Introduction

The auditory system relies on a cochlear amplification process to achieve its high dynamic range and sharp frequency selectivity. The inner ear hair cell hair bundle is comprised of an array of graded length stereocilia organized in a staircase manner. The hair bundle is hypothesized to be an active force generator and integral to cochlear amplification (Howard and Hudspeth, 1987; Kennedy et al., 2005; Beurg et al., 2008; Hudspeth, 2008). Quantifying the mechanical properties of the hair bundle is essential to evaluating its role in cochlear amplification.

Hair bundle arrays in different species and organs vary in height, number of rows, stereocilia thickness, staircase step size, and coherence, defined here as how uniformly and reproducibly stereocilia will move relative to each other in time, direction and distance. Mammalian cochlear hair bundles are unique in having fewer stereocilia rows, typically three, with the lower two rows possessing functional mechano-electric transduction (MET) channels (Beurg et al., 2009). The stereocilia of low-frequency hair bundles are highly synchronous, where individual stereocilia move in unison in time, distance, and direction (Kozlov et al., 2007; Karavitaki and Corey, 2010). Mammalian cochlear bundles lack coherence (Langer et al., 2001; Nam et al., 2015; Scharr and Ricci, 2018). Importantly, this asynchrony of stereocilia motion can alter the macroscopic manifestations of channel gating and adaptation (Nam et al., 2015). Reduced coherence makes hair bundles more susceptible to the mode of stimulation, as the bundles can conform to the temporal and spatial variations in the stimulus. As hair bundles are stimulated in a variety of manners *in vivo*, from free standing to embedded in an overlying membrane and from sinusoidal modulation to static displacement, bundle coherence is important in shaping how these stimulations are translated to a force sensed by the MET channel. Thus, understanding the within bundle and between stereocilia variations in movement is critical to constructing the force sensed by the MET channels which cumulatively generates the receptor current.

Two stimulations methods are most used in *ex vivo* experiments to interrogate the MET process: stiff probes and fluid jets. For mammalian hair bundles, stiff probes are susceptible to uneven stimulation of the hair bundle due to the decreased bundle coherence (Nam et al., 2015). On the other hand, the fluid jet is argued to provide a more uniform hair bundle stimulation (Corns et al., 2014); however, details of the hair bundle motion’s spatial uniformity have not been described. Using high-speed imaging of the hair bundle during fluid-jet stimulation, we characterize the motion of the whole outer hair cell hair bundle. We found that hair bundles exhibit a spatial and temporal non-uniformity when stimulated with a fluid jet. The spatial non-uniformity is contributed to by hair bundles acting as a barrier to fluid flow to alter the flow around the bundle and the presence of lateral links that redistribute forces within the hair bundle. We found the temporal non-uniformity, which appears as a slower motion in the direction of the original stimulus (creep), results from linear passive mechanics intrinsic to the hair bundle, which is consistent with two viscoelastic-like mechanisms present in the hair bundle.

## Results

### Spatially Non-uniform Hair Bundle Displacement

The fluid jet is a common technique used to stimulate hair bundles in *ex vivo* hair cell experiments (Kros et al., 2002; Dinklo et al., 2007; Corns et al., 2014). The fluid jet ejects fluid from a pipette tip causing a fluid velocity that exerts a drag force on the hair bundle (Dinklo et al., 2007). Using a force step hair bundle stimulation reveals underlying mechanical properties of the hair bundle (Crawford and Fettiplace, 1985; Howard and Hudspeth, 1987; Ricci et al., 2000; Kros et al., 2002; Kennedy et al., 2005; Beurg et al., 2008). The stereocilia displacement with fluid jet stimulation will vary with both intrinsic hair bundle properties (e.g., individual stereocilia stiffness, the position of the stereocilia with respect to the fluid flow, the stereocilia height, and the hair bundle coherence which describes how well coupled the stereocilia are to each other) and fluid jet construction and position (e.g., the size of the fluid jet pipette tip opening, the shape of the fluid jet pipette taper, the angle of the fluid jet in *z* with respect to the hair bundle, the lateral and vertical position of the fluid jet relative to the hair bundle, properties of the piezoelectric actuator, and load on the piezoelectric actuator). Previous experiments measured hair bundle movement with a dual photodiode system (Corns et al., 2014, 2016; Beurg et al., 2015; Tobin et al., 2019). This system can only measure the displacement of a portion of the hair bundle in a single direction and its sensitivity will be directly related to the intensity and contrast of the hair bundle image being projected (Crawford and Fettiplace, 1985; Geleoc et al., 1997). The lack of cohesion in mammalian hair bundles (Langer et al., 2001; Nam et al., 2015) makes it necessary to simultaneously monitor motion at multiple positions along the hair bundle to identify spatial non-uniformities. Our high-speed imaging system allowed for these investigations (Caprara et al., 2019).

**FIGURE 1:**
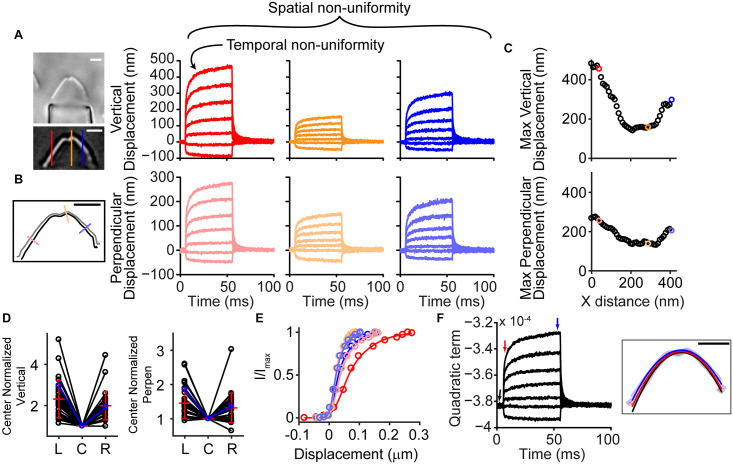
Hair bundle motion was not spatially uniform with a fluid jet stimulus. **(A)** Hair bundle images show the raw image (top) and a filtered image used for analysis with colored lines indicating where the vertical motion was measured. Plots of the vertical hair bundle motion at three different positions along the hair bundle indicated differing movement magnitudes demonstrating the spatial non-uniformity. Within each displacement trace there was a temporal non-uniformity exhibited as a creep of the hair bundle. **(B)** Perpendicular hair bundle motion was calculated at different points along the hair bundle (colored lines) and showed different movement magnitudes. The image showed the resting position of the hair bundle in black, with the position at the end of the force step shown in gray. Corresponding displacements along the perpendicular lines are color coded. **(C)** Maximum ending displacements for the cell in panels **(A,B)** as a function of x distance along the hair bundle, indicating that the positions toward the sides of the hair bundle move a greater distance. **(D)** Summary of 29 cells analyzed showing the center normalized movement of the left (L), center (C), and right (R) side of the hair bundle for the vertical and perpendicular directions. The cell in panel **(A–C)** is highlighted in blue. **(E)** The possible resulting activation curves due to the motion at different parts of the hair bundle indicated that the parameters differed more than 2x depending on where and how the motion was measured. Activation curves were generated with the displacement at the time of the peak current. **(F)** Fitting a quadratic equation to the hair bundle position in each frame indicated changes in the hair bundle width that follows a similar time course as the motion in panel **(A,B)** and further confirmed the larger movements toward the hair bundle sides. Right schematic shows the position of the hair bundle at each point along the hair bundle (circles) and the resulting quadratic equation fit (lines). Different colors represent different times when the hair bundle position and quadratic fits were obtained according to the colored arrows in the graph. Scale bars = 2 μm.

Stimulated OHC hair bundles consistently showed a spatially non-uniform motion, where the hair bundle edges moved outward and upward more readily than the hair bundle center (Supplementary Video 1). Taking advantage of high-speed imaging allowed tracking of nanometer scale deflections of the entire hair bundle. Using techniques of position tracking of the hair bundle with fitting the filtered hair bundle intensity profile with a Gaussian curve developed for particle tracking (Yildiz et al., 2004; Ramunno-Johnson et al., 2009; Isojima et al., 2016), we could achieve sub-pixel and sub-diffraction limit displacement of the hair bundle. To determine how the entire hair bundle moved, we calculated the position of the hair bundle in each column of the hair bundle image for every frame of the stimulation movie. When looking at the vertical motion of the hair bundle (i.e., columns of the image), the sides of the hair bundle moved more than the center (Figure 1A). We also observed that the sides moved more laterally than the center. We are not able to track the movement of individual stereocilia in OHC hair bundles due to resolution limitations; therefore, when using a vertical line on the hair bundle contour, the lateral motion can overestimate the displacement measured on the sides of the hair bundle. The overestimate is due to the stationary line where the displacement is measured sliding to more medially positioned stereocilia along the hair bundle between the resting and the stimulated positions when lateral stereocilia movement is present. To diminish the potential artifact and ensure that the spatial non-uniformity was not a function of the measurement, we analyzed the motion in the direction perpendicular to the hair bundle contour (Figure 1B). With both analysis techniques, the middle of the hair bundle moved less than the sides (Figures 1C,D). Overall, the OHC bundle appeared to broaden the width of its V-shape upon stimulation. Using a third strategy to describe the bundle motion, we fit a quadratic equation to the hair bundle. The quadratic coefficient, which described the steepness of the parabola, indicated a flattening curve over the course of the stimulation (Figure 1F), consistent with greater lateral motion at the edges.

The spatially non-uniform motion altered how activation curves were represented. Activation curves generated with the fluid jet tend to be quite steep as compared to those generated with a stiff probe (Fettiplace and Kim, 2014). This discrepancy in part depends on where the bundle motion is measured. Since hair bundle motion magnitude varies with position along the hair bundle, the resulting activation curves can differ by more than two-fold (Figure 1E).

**FIGURE 2:**
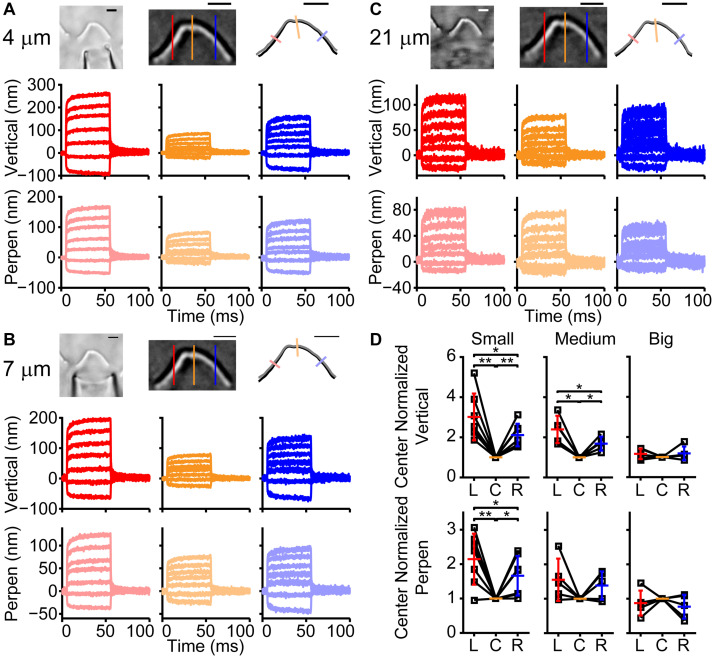
Effect of fluid jet size on hair bundle motion on the same fixed hair bundle. **(A)** Raw (left) and filtered (middle) hair bundle images are shown with a small pipette fluid jet (4 μm). The calculated hair bundle position with the corresponding perpendicular lines is shown on the right. Darker lines represent the vertical hair bundle motion at corresponding positions in the filtered image. Lighter lines indicate the perpendicular hair bundle motion along the lines in the right image. **(B,C)** The same analysis as in panel **(A)** is shown, but with a medium-sized pipette (7 μm wide) in **B** and a large fluid jet (21 μm wide) in **C**. Stimulus size was adjusted to yield comparable center bundle motion. **(D)** Summary of hair bundle motion amplitude for the largest step size with left (L) and right (R) side motion normalized to the center (C). Individual cells are shown and demonstrate that the larger pipettes deliver more uniform hair bundle motion when analyzing the vertical or perpendicular hair bundle motion. *n* = 7, 5, 5 for small, medium, and large pipettes, respectively. Scale bars = 2 μm. *p < 0.05, **p < 0.01.

Was the spatially non-uniform bundle motion driven by characteristics of the fluid jet or by characteristics of the hair bundle? Fluid flow from the fluid jet pipette center will be greatest with flow velocity decreasing toward the edge of the cone-shaped flow pattern (Tobin et al., 2019). To better understand the fluid flow role in shaping the observed hair bundle motion, the fluid-jet pipette size was varied. For these experiments, we used fixed tissue where only passive hair bundle properties are present, and multiple fluid-jet pipette sizes could easily be tested on the same bundle (Figure 2). Examples of small (4 μm), medium (7 μm), and large (21 μm) diameter pipettes showed spatially non-uniform displacement (Figure 2). Smaller pipette diameters created more hair bundle edge motion than larger pipettes. Larger pipette diameters resulted in the most spatially uniform displacement; however, the largest pipettes elicited a visually noticeable movement of the underlying epithelium (data not shown). Increasing pipette size increases the center area with a more uniform fluid velocity (Tobin et al., 2019). Thus, it makes sense that the larger pipette results in more uniform stimulation. However, small pipettes would have lower fluid velocity toward the edge of the hair bundle, which is predicted to result in smaller edge stimulation, but instead, we observed larger displacements suggesting that more than simple uniformity of flow is needed to explain the results.

### Hair Bundle Disrupts the Fluid Flow

To understand the apparent contradiction between the bundle motion and the spatial distribution of fluid velocity, we postulated that the flow disruption by the hair bundle may contribute to the spatially non-uniform motion. To better assess fluid flow from the fluid jet and determine how the hair bundle obstructs and modifies fluid flow, we monitored fluid flow using the fluorescent dye Alexa 594 (1 μM), simulated fluid flow from a pipette using a 3-dimensional (3D) model, and measured fluid flow rates around the hair bundle with a flexible glass fiber.

**FIGURE 3:**
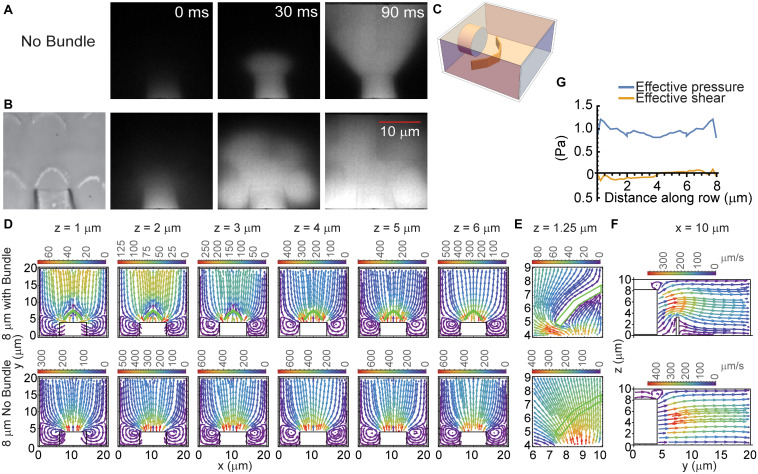
Hair bundle effects on fluid flow. **(A,B)** 1 μM Alexa 594 was used to track the flow from the fluid jet pipette. Fluorescent images at three time points corresponding to before the stimulus (0 ms), immediately after stimulus onset (30 ms), and during the stimulus (90 ms) in the absence **(A)** and presence **(B)** of the hair bundle are shown. **(C)** Schematic of the 3D model structure is shown. **(D)** Provides the FEM computed, steady-state fluid flow from a pipette of 8 μm diameter measured at various distances above the apical surface (z) with (top row) and without the hair bundle (bottom row). The hair bundle is depicted in the green outline. The color of flow lines indicates the fluid flow rate according to the color scale above the plot. **(E)** Presents an enlargement of half the bundle to better show how fluid flow was impeded by the hair bundle at *z* = 1.25 μm. **(F)** Provides a side view of the fluid flow with (top) and without (bottom) the hair bundle at the 10 μm x position from panel **(D)**. **(G)** Presents the effective pressure and the effective shear across the face of the hair bundle. Values are plotted against the distance along the hair bundle contour.

Imaging fluid flow in the presence of the hair bundle demonstrated that the hair bundle acted as a barrier to fluid flow (Figures 3A,B). Fluid flow from the pipette in the absence of the hair bundle resulted in the typical cone-shaped flow (Figure 3A). When the hair bundle was present, the flow was disrupted and appeared to create billows of dye (Figure 3B). The dye appears to deflect backward and toward the hair bundle sides and move around and over the hair bundle rather than through it. To better assess the hair bundle impact on fluid flow from the fluid jet, a steady-state 3D model was generated where a pipette of medium diameter (8 μm) was placed in front of the 4 μm tall hair bundle (Figure 3C). The hair bundle was modeled as panels simply using rootlet stiffness as the primary source of stiffness; top connectors, lateral links, multiple stereocilia rows, and tip links were not included. In the presence of the hair bundle, fluid flowed around and over the hair bundle (Figures 3D–F). Fluid flow rates were considerably higher in the region in front of the fluid jet when the hair bundle was not present (Figure 3D). Fluid flow rates slowed with distance away from the pipette when no bundle was present (Figure 3D, bottom row). When the hair bundle is present, flow increased at further distances from the pipette (Figure 3D, *z* = 1–3 μm) due to flow coming from higher *z* planes as seen in the side views of fluid flow (Figure 3F). Zooming in on one half of the bundle (Figure 3E) showed how the flow was hindered by the hair bundle presence, such that flow was more outward (lateral) at the bundle edge and more inward (medial) at the bundle center. The effective pressure (force perpendicular to the hair bundle contour) supports the larger displacements measured at the hair bundle edges (Figure 3G). The effective shear (forces parallel with the hair bundle contour) was small, indicating the predominant force is perpendicular to the hair bundle contour. This model showed that the interaction between the hair bundle shape and the fluid flow differentially altered the force on the hair bundle with greater forces on the edges of the hair bundle as compared to the center even when the starting fluid flow was uniform when coming out of the stimulating pipette.

**FIGURE 4:**
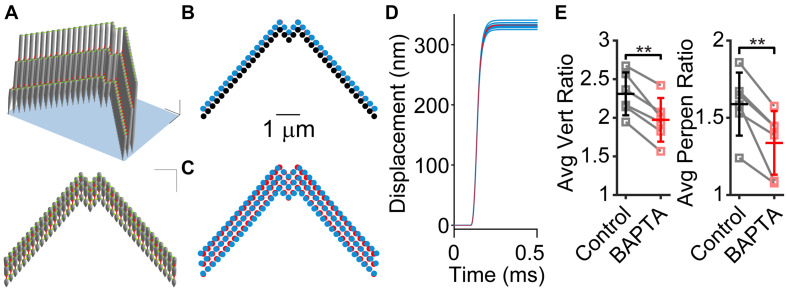
Stereocilia displacement non-uniformity is greater when lateral links are present. **(A)** 3D model of stereocilia hair bundle is shown in two perspectives with tip-links shown in red and lateral links in green. **(B)** Movement of the tallest row of stereocilia from the rest position (black) to the final position when tip links are present with (blue) and without (red) lateral links. Note that the blue almost entirely covers the red stereocilia. **(C)** Position of stereocilia in the entire bundle that is multiplied by five times to visualize the difference between the presence (blue) and absence (red) of lateral links. **(D)** Plots of the displacement of individual stereocilia showing that when lateral links are present (blue traces) there is a distribution of displacement sizes, whereas when lateral links are absent, displacement sizes are the same for each stereocilium (red traces). **(E)** Re-analyzing experiments in Caprara et al. (2020) with BAPTA treatment of hair bundles show that after BAPTA treatment, hair bundle non-uniformity is decreased. The center (C) normalized vertical ratios on the left (L) and right (R) side of the hair bundle were averaged for stimulations before (control) and after BAPTA treatment in the same cells. For controls, the largest stimulation was used and after BAPTA treatment, we used the closest matched center displacement stimulation for the calculation. We preformed this analysis using both vertical motion and perpendicular motion like Figures 1A,B (*n* = 6, Vert, *p* = 0.0011; Perpen, *p* = 0.0050). **p < 0.01.

To understand the effects of tip links, lateral links, and hair bundle splaying, we used a finite element stereocilia model populated with tip links and lateral links (Figure 4A; Nam et al., 2015). We modeled a force step stimulation applied to only the tallest stereocilia row to investigate the effects of links on the hair bundle motion. We found that the presence of lateral links within the hair bundle contributed to a small difference in motion. When lateral links were present in the model (Figures 4B–D, blue) the hair bundle exhibited splaying, where the edges of the hair bundle moved a larger distance than when lateral links were absent, indicating that the links redistributed the forces on the stereocilia (Figures 4B–D, red). However, the difference in displacement of each stereocilium was small, only differing by 5.5% (Figure 4C). The stimulated positions of the hair bundle indicate that the lateral links had a small contribution to the hair bundle splay (Figure 4D).

Previously, we found that BAPTA treatment of the hair bundle resulted in changes to the creep behavior (temporal non-uniformity, Figure 1A), but the creep remained, implicating a non-BAPTA-sensitive structural contributor (Caprara et al., 2020). Using the new analysis method to characterize the motion of the whole hair bundle, we also observed that the spatial non-uniformity was decreased after BAPTA treatment (Figure 4E), indicating that the BAPTA-sensitive links had a contribution to the spatial non-uniformity, consistent with the modeling data.

**FIGURE 5:**
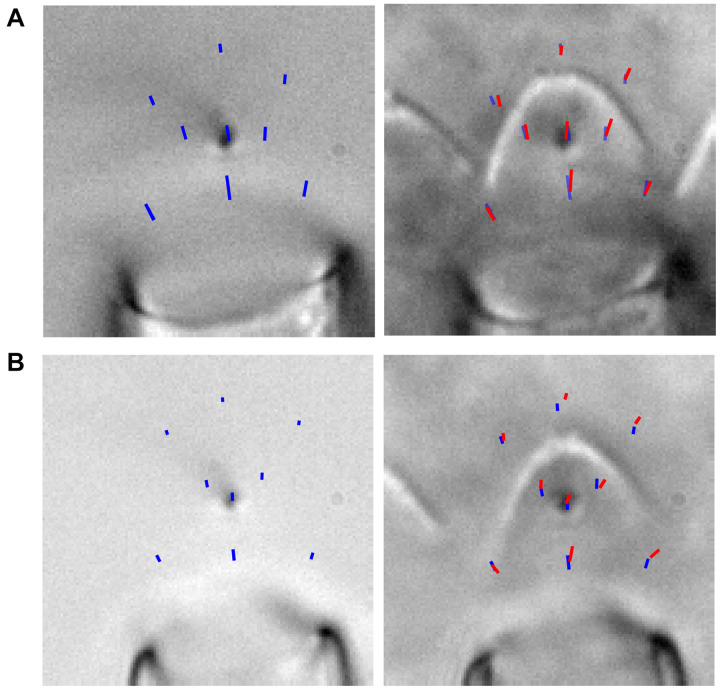
**(A,B)** Two examples of fluid forces with (red) and without (blue) the hair bundle are shown. The images show the trajectories (from rest state to steady-state position) of a flexible fiber bent down toward the tissue such that it appeared as a point in *x-y* and a line in *x-z*. The fiber was moved to various positions, and the trajectory of four presentations of the largest stimulation at each position is overlaid on the image. This demonstrated that the hair bundle presence altered the flow, and the variability between stimulations of a given fiber position was very low. For experiments without the hair bundle, the fluid jet and flexible fiber were moved ∼200 μm above the epithelium.

A third approach to determine whether bundle motion reflected stimulus anomalies was to use a flexible fiber to monitor the force delivered by the fluid jet at different positions around the hair bundle (Figure 5). To investigate the potential contribution of lateral forces on the hair bundle, we performed experiments with glass fibers bent such that they appeared as a point in the image and extended upward in the *z* direction. This arrangement would provide information about forces in *x-y*, similar to that described with the modeling in Figure 3F. The flexible fiber data had a caveat that the total motion observed was due to a weighted sum of the forces along the length of the fiber, with forces at the end of the fiber weighted more heavily. Thus, some of the observed motion was due to fluid-flow forces on the fiber above the bundle. The fluid flow above the hair bundle would lead to an underestimate of the hair bundle effects on the flow, since fluid flow above the hair bundle is largely unchanged by the hair bundle based on the 3D model (Figure 3D). Two examples of fluid-jet flow in the absence (blue) and presence (red) of the hair bundle showed that the hair bundle presence altered the fluid flow (Figure 5). The length of the plotted lines indicates the distance traveled for a saturating MET stimulus. Four trajectories were plotted for each position corresponding to four presentations of the same stimulus size. Trajectories appeared to be a single line due to the highly reproducible movement of the fiber for each presentation. In these examples, the forces were mainly rotated toward the right due to the hair bundle presence. A significant difference in the fluid drag force is apparent at different distances of the fiber (Figure 5, blue). This decrease in drag force is consistent with the results of the 3D model (Figures 3D,E) and previous results (Tobin et al., 2019). These data were like the predictions of the model (Figure 3F) where the flow was altered in the presence of the hair bundle, but detailed differences in fluid forces may have been caused by hair bundle and fluid jet orientation differences in the experiments. The experimental data further confirmed that the spatially non-uniform hair bundle motion was due to fluid dynamics of the fluid jet with the hair bundle acting as a barrier.

### Mechanical Creep Is Linearly Related to Stimulus Size

**FIGURE 6:**
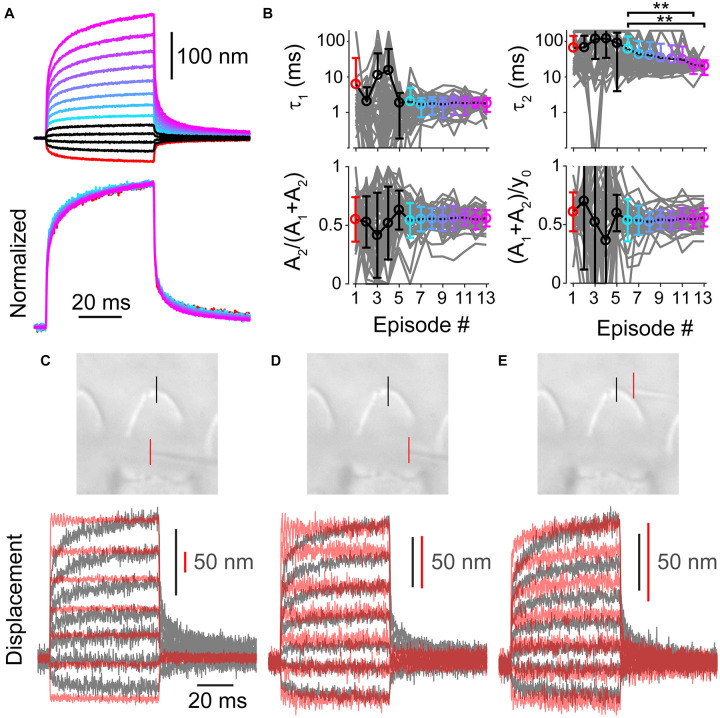
**(A)** Average of all measured displacements and below are those traces normalized to the end of 50 ms force step (*n* = 47). Episode number color coded according to panel **(B)**. Only episodes 1 and 6–13 are shown for the normalized traces, others are omitted due to low signal to noise. **(B)** Fit parameters for creep vs. stimulation episode number with 1 mM BAPTA intracellular buffer in OHCs. Each cell is plotted in gray with mean ± standard deviation. Tukey-Kramer *post hoc* analysis on τ_2_ showed significance for only the comparisons shown; **p < 0.01. **(C–E)** Force measurements around the hair bundle indicate that the hair bundle creep is not due to fluid jet anomalies. **(C)** Image of the fiber and hair bundle stimulated by a fluid jet is shown with locations of motion measurement indicated by colored lines. Measured bundle (black) and fiber (red) displacement is plotted below. Resonance observed in the fiber motion was likely due to the vibration of the fluid-jet pipette observed with larger stimulations. **(D)** Shows force out of the fluid jet was square as indicated by the fiber motion in front of the hair bundle toward the side. **(E)** Motion of the of fiber and bundle when the fiber was located behind the hair bundle is shown. When the fiber was behind the hair bundle side, the bundle blocked the flow causing a slower fiber onset similar to the hair bundle creep.

Up until now, we have investigated the source of the spatial non-uniformity of the hair bundle stimulation. When using a step-like force stimulation, the hair bundle displacement also exhibits a temporal non-uniformity that is characterized as a mechanical creep (Howard and Hudspeth, 1987; Russell et al., 1992; Kennedy et al., 2005; Beurg et al., 2008; Caprara et al., 2019). For a given hair bundle, the mechanical creep was linearly related to the stimulation voltage, such that the contribution of the creep components [A_2_/(A_1_+A_2_)] and the relative magnitude of the creep to the total displacement of the bundle during the 50 ms force step [(A_1_+A_2_)/y_0_] remained constant with stimulus episode (Figures 6A,B). Hair cells were patch-clamped with 1 mM BAPTA as the intracellular calcium buffer and subjected to 13 stimulation intensities (*n* = 47). We fit the mechanical creep after the force had plateaued (Caprara et al., 2019) with a double exponential equation (see section “Materials and Methods”). Stimulation episode 1 is the largest negative stimulation and 3 is the smallest negative stimulation. Episode 4 is the smallest positive stimulation and 13 is the largest positive stimulation. Looking at the data from episodes 6–13, where variability is lower due to greater signal to noise in the fitting, shows that the relative magnitude of the slow creep [A_2_/(A_1_+A_2_)] and the relative magnitude of the total creep [(A_1_+A_2_)/y_0_] was constant (Figure 6B, *p* = 0.95 and *p* = 0.76, respectively). The time constant τ_1_ was also constant, but τ_2_ was not (1-way ANOVA *p* = 0.87 and *p* = 0.0014, respectively). That the stimulations had comparable relative creep magnitudes supports a linear process underlying the creep.

Even though the creep is a linear process, we wanted to distinguish whether the creep was due to characteristics of the fluid jet or the hair bundle. We measured the kinetics of a flexible fiber motion placed around the hair bundle (Figures 6C–E). The flexible fiber allowed us to visualize fluid kinetics that the 3D steady-state model did not provide. Fiber placement in front of the hair bundle indicated a step-like force stimulus across the hair bundle face (Figures 6C,D). Even when the fluid drag force due to the fluid jet was step-like, the hair bundle creep was observed, supporting a conclusion that the creep was a result of the hair bundle’s mechanical properties. The force on the fiber behind the bundle exhibited a creep similar to that observed by the hair bundle (Figure 6E). This indicates that the force kinetics were altered by the hair bundle potentially acting as a barrier to the flow, which slowed the equilibration of force behind the bundle. These data confirmed that the fluid-jet stimulator itself is not responsible for the hair bundle creep.

## Discussion

Fluid jet force stimulation of the mammalian outer hair cell bundle leads to both spatial and temporal non-uniformities. The mammalian hair bundle is incoherent, which allows for different parts of the hair bundle to have different displacement magnitudes with fluid-jet stimulation. Using a combination of experimental manipulations and mathematical modeling, we found that the spatially non-uniform motion have contributions from altered fluid dynamics imparted by the hair bundle acting as a barrier to the fluid flow and by linkages within the hair bundle distributing the fluid forces. For the temporal non-linearity that is exhibited as a creep in the hair bundle, the creep is due to characteristics of the hair bundle and appear to result from a passive linear system.

### Spatially Non-uniform Hair Bundle Motion

Fluid flow from our fluid jet has a typical cone shaped flow and produces a step-like drag force, but the flow is impacted by the apical surface, the hair bundle, and stimulus intensity. This, coupled with a previously documented lack of cohesiveness within the bundle (Langer et al., 2001; Nam et al., 2015), leads to stereocilia motion that is spatially non-uniform. Experimental and modeling data demonstrate that the hair bundle acts as a physical barrier to fluid flow, altering flow around the hair bundle and contributing to the spatial non-uniformity of hair bundle displacement.

OHC bundle motion displays a spatial non-uniformity with a broadening of the bundle shape during stimulation such that the bundle edges are moving laterally as well as forward. This characteristic OHC hair bundle motion is due to the complex fluid dynamics that the hair bundle barrier creates through the inherent shape and architecture of the OHC hair bundle. OHCs are thought to be stimulated by basilar membrane motion shearing the hair bundle embedded in the tectorial membrane, therefore, the motion induced by a fluid jet is unlikely to mimic an *in vivo* response. However, it does suggest that the lack of hair bundle coherence allows the hair bundle to follow the temporal and spatial components of the stimulus more than a coherent bundle would, and this motion would shape the receptor potential. Since the OHC bundles are embedded in the tectorial membrane (Lim, 1972), the stiffness of the tectorial membrane will be critical in determining how the hair bundle moves.

An alternative stimulation mode of the hair bundle could be to “squish” the bundle, which could lead to hair bundle splay like we observe with fluid jet stimulation. This idea is supported by the orientation of tip-links in OHCs that can be more laterally oriented on the sides of the hair bundle when we inspect some scanning electron micrographs of the hair bundle (Velez-Ortega et al., 2017), suggesting that they may be sensitive to hair bundle splay. Additionally, evidence exists for changes in the distance between the tectorial membrane and surface of the epithelium (reticular lamina), which would also be consistent with the “squish” mode of stimulation (Guinan, 2012; Hakizimana et al., 2012; Lee et al., 2015). To be clear, the fluid jet does not replicate *in vivo* stimulation and care must be taken with interpreting these bundle motions as being physiologically relevant. Our data simply points out the need for a better understanding of *in vivo* bundle motion, because the motion will directly shape the receptor current.

### *Ex vivo* Stimulation Methods Are Imperfect

The stiff probe and fluid jet are the predominant methods of *ex vivo* hair bundle stimulation. The stiff probe is fast (as fast as 11 μs rise time) (Peng et al., 2013), mechanically loads the hair bundle (the probe touches multiple stereocilia), does not contact all stereocilia (Nam et al., 2015), and delivers a displacement stimulus that synchronizes a set of stereocilia. The fluid jet is slow (500 μs rise time) (Caprara et al., 2019), does not load the hair bundle (i.e., does not touch the hair bundle), can stimulate all stereocilia (albeit at different axes depending on hair bundle morphology), and delivers a force stimulus. With both stimulus types, we do not know how the second or third stereocilia rows (middle and shortest) are moving, for example the second row stereocilia might be directly pushed into the first row or it could be pulled by the movement of the first row. The differences in stimulus mode shape the MET responses differently (Caprara et al., 2019). It is debated whether stiff probes or fluid jets represent a more physiological stimulation (Corns et al., 2014; Fettiplace and Kim, 2014). Data presented here identify issues with the fluid jet that make it unlikely to represent a physiological stimulation to either outer or inner hair cells. Problems also exist with the stiff probe (Nam et al., 2015); therefore neither device likely represents a physiological stimulus. However, each has biophysical advantages and disadvantages. Given the major differences in hair bundle response and channel activation associated with the mode of stimulation, it now becomes critically important to develop more physiological stimuli to assess the *in vivo* MET current properties accurately. One strategy is to identify how the hair bundle moves *in situ* and develop a device to mimic that. OHC hair bundles have long been hypothesized to be embedded in the tectorial membrane overlying the hair bundles (Kimura, 1966; Lim, 1972), but old and recent data also indicate that the IHC hair bundle may be attached to the tectorial membrane as well (Hoshino, 1976; Hakizimana and Fridberger, 2021). Knowing there are connections is not enough to mimic stimulation, as the mechanical properties of this coupling will be critical in hair bundles where the stereocilia are not strongly coupled. A better stimulation technique may be to keep the tectorial membrane intact or create an artificial tectorial membrane and stimulate the membrane directly. One potential flaw in these approaches may be that *in vivo*, it is the basilar membrane that is moving. Previous hemi-cochlea techniques achieve basilar membrane stimulation and may represent the most physiological stimulation to date (He et al., 2004).

### Origins of the Temporal Non-uniformity

The bundle displacement shows an initial motion due to the stimulus force onset, followed by a mechanical creep defined as a continued motion in the direction of the stimulus. The mechanical creep was characterized by a fast and slow exponential process and was not due to the motion of the apical surface (Caprara et al., 2019) and is diminished by fixing the tissue (Caprara et al., 2020). BAPTA treatment increased the overall motion and altered the creep kinetics but did not eliminate it (Caprara et al., 2020), suggesting that channel gating or tip-link stretch do not solely underlie the creep but may contribute to it. This result was consistent with Tobin et al. (2019), where the creep was not abolished after BAPTA treatment for the 2 and 4 kHz locations. When calculating the relative softening like Tobin et al. (2019) using time windows of 8–10 ms and 48–50 ms after stimulus onset, the hair bundle observes a relative softening of 17.1 ± 3.6% before and 9.5 ± 4.4% after BAPTA treatment (*n* = 6, *p* = 0.0012), similar to values of Tobin et al. (2019). Since the creep is not lost at other positions indicates that the slow creep is not simply determined by the tip-link and/or MET processes.

**FIGURE 7:**
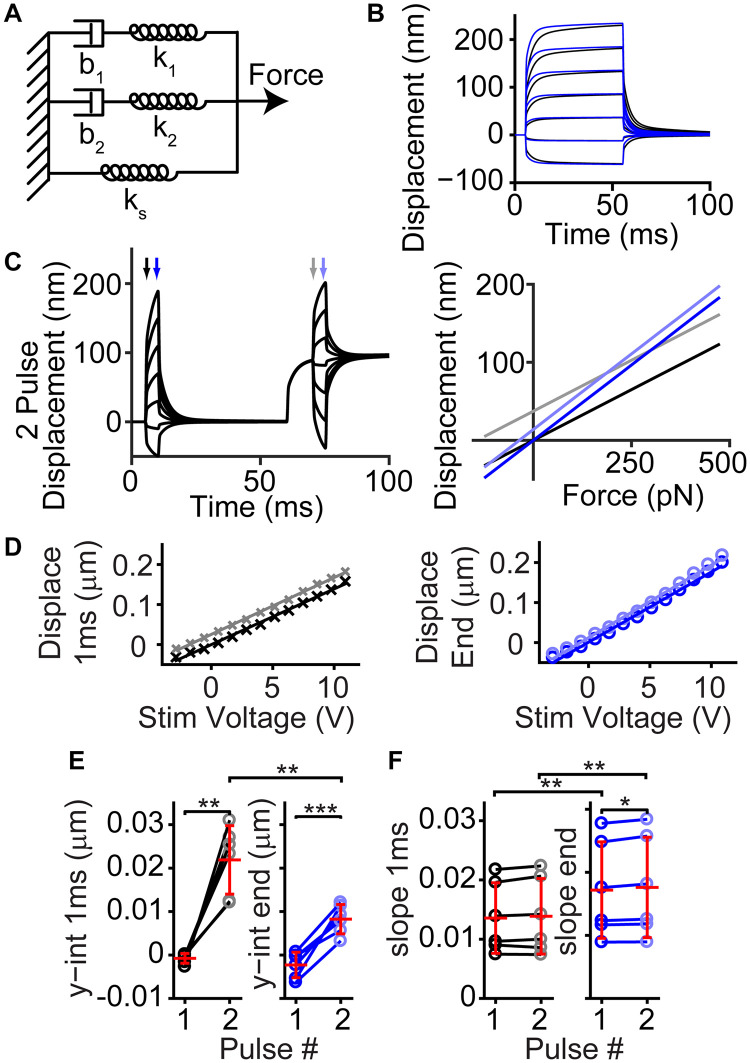
Simple passive linear mechanical model explained the general shape of the complex hair bundle motion. **(A)** Passive mechanical model with a combination of springs (k) and dashpots (b) mimicking the classic model of motor adaptation with a second spring-dashpot combination to add the second creep time constant observed in hair bundle motion is shown. **(B)** Black lines are the resulting motion based on the model in panel **(A)** using 100 pN step sizes starting at –125 pN, blue lines indicate the resultant motion when k_*s*_ and force applied is increased 2-fold, resulting in a similar effect as seen with fixed tissue (Caprara et al., 2020). **(C)** Using the same linear model, a 2-pulse experiment resulted in similar bundle motions (left) and changes in slope and intercept as observed in panel **(D)**. Displacement vs. force lines were taken at timepoints indicated by arrows in the displacement graph. **(D)** Plots of the bundle displacement at 1 ms and 3.9 ms (near the end of the stimulus) after stimulus onset for –84 mV data (analyzed from Caprara et al. (2019) Figure 3A) show a roughly linear relationship with fluid-jet stimulus voltage. Linear fits of the data indicate the slope (indicator for the effective stiffness of the hair bundle) and y-intercept (indicator of the offset of the hair bundle). **(E)** Summary of the y-intercepts indicated a positive shift in the hair bundle displacement with stimulation size between the first and second activation curves supporting the presence of a mechanical dashpot (*n* = 6, y-int 1 ms, *p* = 0.0015; y-int end, *p* = 0.00094). **(F)** Changes in the slopes of the fit indicated little change between pulses at the same time point, but a clear difference between the beginning and end of the short activation curve pulse indicating a change in effective stiffness (*n* = 6, Pulse 1, *p* = 0.0040, Cohen’s *d* = 0.53; Pulse 2, *p* = 0.0036, Cohen’s *d* = 0.52). *p < 0.05, **p < 0.01, and ***p < 0.001.

We modeled the hair bundle creep using a simple combination of springs and dashpots (Figure 7A). Past models of how a fluid jet stimulates the hair bundle suggested a slow creep is manifested through a spring-dashpot series combination (Vollrath and Eatock, 2003). We observe a similar kinetic, except with a double exponential onset, therefore warranting a second spring dashpot series combination. A viscoelastic material can be described by a similar spring-dashpot series combination. This model recapitulates the creep observed by the hair bundle and the effects of tissue fixation (Figure 7B). Using the model, we can also predict what would happen during a 2-pulse stimulation (Figure 7C). We analyzed our previous data (Caprara et al., 2019), and we found a similar behavior (Figures 7D–F). When increasing the stereocilia pivot stiffness (k_*s*_), we could mimic the changes observed with tissue fixation (Caprara et al., 2020). Since tissue fixation will remove any active processes, the resultant creep arises from a linear passive system. Fluid dynamics are unlikely to account for all of the observed creep, since force out of the pipette is step-like (Figure 6C,D), and similar creep is observed with flexible fiber stimulations where fluid dynamics are unlikely to have a role (Russell et al., 1992; Kennedy et al., 2005; Beurg et al., 2008). There are a few potential origins of the viscoelastic-like mechanism. One possibility is that diffusion of the lipid bilayer plays an important role (George et al., 2020), since the spring-dashpot model provides an approximation to the diffusion process. BAPTA insensitive hair bundle links may provide viscoelastic elements that are stretched during bending at the stereocilia base to generate the observed passive mechanics. These viscoelastic elements could also exist in the stereocilia rootlet, where the bending occurs (Furness et al., 2008; Kitajiri et al., 2010). More studies are required to narrow the list of potential origins.

Previously, the slow creep component was thought to originate from the slow adaptation mechanism and was modeled as a viscoelastic type mechanism (Howard and Hudspeth, 1987; Caprara et al., 2020). Even though the model contains only passive components of springs and dashpots, active motor molecules (myosins) were responsible for the dashpot. When using fixed tissue, the active motor molecule cannot be functional, therefore the presence of the creep when the tissue is fixed also argues against the motor model of adaptation underlying the slow creep component in OHCs (Caprara et al., 2020). Although our modeling of increasing the stereocilia pivot stiffness was able to recapitulate features of the fixed tissue motion, we cannot completely rule out that the overall diminished creep with tissue fixation was not just due to decreased function of the myosin motor in the viscoelastic-like element. However, this is rather unlikely, since the system appeared quite linear with negative, positive, small, and large stimulations resulting in similar creep properties in live tissue (Figures 6A,B). In the motor model of adaptation, larger stimulations would lead to saturation of slow adaptation and would be correlated with a decrease in the slow creep component, but we do not observe this effect (Howard and Hudspeth, 1987; Hacohen et al., 1989; Assad and Corey, 1992). Thus, the more likely scenario is that the creep is a result of a linear passive system.

### Physiological Relevance of Hair Bundle Motion

There are a few reasons for understanding the hair bundle motion. Since the hair bundle is composed of stereocilia shear detectors all along the hair bundle, if different parts of the hair bundle have different displacement sizes, then the activation curve and hence sensitivity of the hair bundle is broadened (Nam et al., 2015). When trying to understand the sensitivity of the cochlea and the mechanisms that contribute to sensitivity, it is imperative to know how the hair bundle is moving using the different stimulus modalities. This allows the proper biophysical characterization of the MET process itself. The next step is to determine the movement of the hair bundle *in vivo*. How the hair bundle moves in vivo is important for understanding the encoding of stimuli in the cochlea. The mechanical properties of the tectorial membrane and its interaction with the hair bundle as well as how the hair bundle is stimulated (shear vs. squish), can alter the coding of the stimuli. If these mechanical properties suggest a force stimulation on the hair bundle rather than the commonly thought of displacement stimulus, then the mechanical properties of the hair bundle will also alter coding of stimuli. For instance, the mechanical creep exhibited by the hair bundle during force stimuli can prolong the receptor current, which may increase fidelity of stimulus detection.

## Conclusion

Hair bundles stimulated with a fluid jet force stimulation exhibit spatial non-uniformities due to the hair bundle modifying the fluid flow and the inherent connections within the hair bundle that redistribute the forces on the stereocilia leading to the sides of the hair bundle exhibiting greater displacements than the center of the hair bundle. The bundle also exhibits a temporal non-uniformity seen as a mechanical creep that can be described by a linear passive system containing two viscoelastic-like mechanisms.

## Materials and Methods

### Preparation and Recordings

Animals were euthanized by decapitation using methods approved by the University of Colorado IACUC or the Stanford University Administrative Panel on Laboratory Animal Care. Organs of Corti were dissected from postnatal day (P) 6–9 Sprague-Dawley rats (large majority of experiments used P7-P8) of either sex and placed in recording chambers as previously described (Peng et al., 2013). Tissue was viewed using a 100x (1.0 NA, Olympus, Pittsburg, PA, United States) water-immersion objective with a Phantom Miro 320s (Vision Research, Wayne, NJ, United States) camera on a Slicescope (Scientifica, Clarksburg, NJ, United States) illuminated with a TLED+ 525 nm LED (Sutter Instruments, Novato, CA, United States). Tissue was dissected and perfused with an extracellular solution containing (in mM): 140 NaCl, 2 KCl, 2 CaCl_2_, 2 MgCl_2_, 10 HEPES, 2 Creatine monohydrate, 2 Na-pyruvate, 2 ascorbic acid, 6 dextrose, pH = 7.4, 300–310 mOsm. In addition, an apical perfusion, with pipettes tip sizes of 150–300 μm, provided local perfusion to the hair bundles that was often turned off once a giga-ohm seal was achieved. In all preparations, the tectorial membrane was peeled off the tissue. For experiments with fixed tissue, fixation was with a 4% paraformaldehyde in phosphate-buffered saline overnight at 4°C. The solution was prepared from a 16% paraformaldehyde stock (Electron Microscopy Sciences, Hatfield, PA, United States).

### Electrophysiological Recordings

Whole-cell patch-clamp recordings were achieved on first or second row outer hair cells (OHCs) from middle to apical cochlear turns using an Axon 200B amplifier (Molecular Devices, San Jose, CA, United States) with thick-walled borosilicate patch pipettes (2–6 MΩ) filled with an intracellular solution containing (in mM): 125 CsCl, 3.5 MgCl_2_, 5 ATP, 5 Creatine Phosphate, 10 HEPES, 1 Cesium BAPTA, 3 ascorbic acid, pH = 7.2, 280–290 mOsm. For 0.1 mM and 10 mM BAPTA internal solution, the BAPTA and CsCl concentrations were adjusted accordingly to reach 280–290 mOsm. Experiments were performed at 18–22°C. Whole-cell currents were filtered at 10 kHz and sampled at 0.05–1 MHz using USB-6356 (National Instruments, Austin, TX, United States) controlled by jClamp (SciSoft Company, Ridgefield, CT, United States). Voltages were corrected offline for liquid junction potentials. All experiments used a −84 mV holding potential unless otherwise noted.

### Hair Bundle Stimulation and Motion Recording

Hair bundles are stimulated with a custom designed fluid jet driven by a piezoelectric disk bender (27 mm 4.6 kHz; Murata Electronics 7BB-27-4L0, Nagaokakyo, Japan). The fluid jet chamber was filled with low viscosity silicone oil (317667, Sigma-Aldrich). Thin wall borosilicate pipettes were pulled to tip diameters of 5–20 micrometers, filled with extracellular solution, and mounted in the fluid jet stimulator. The piezo-disk bender was driven by waveforms generated using jClamp, and the signals were filtered using an 8-pole Bessel filter (L8L 90PF, Frequency Devices Inc., Ottawa, IL, United States) at 1 kHz and variably attenuated (PA5, Tucker Davis, Alachua, FL, United States) before being sent to a high voltage/high current amplifier (Crawford Amplifier) to drive the piezo-disk. During stimulations, hair bundle motion videos were taken at 10,000 frames per second using the Phantom Miro 320s. Videos were saved for each stimulation and analyzed offline. Four stimulus presentations were averaged together at each stimulus level unless otherwise stated. In some experiments, a short flexible fiber that was straight or bent 90° to appear as a point in the high-speed videos was used to monitor local force generated by the fluid jet.

### Hair Bundle Motion Analysis

Custom MATLAB (MathWorks, Natick, MA, United States) scripts were used for extraction and analysis of the hair bundle motion. Movie frames were imported into MATLAB, and the hair bundle position was extracted using the center point of a Gaussian fit to a band-pass filtered hair bundle image (Ramunno-Johnson et al., 2009) for a given vertical row of pixels in the image to yield sub-pixel resolution. To assess whole bundle motion, the motion of all vertical lines along the hair bundle as well as the perpendicular motion was measured. For the perpendicular motion, the initial hair bundle position was generated at each pixel location using a Gaussian fit to the vertical pixel intensity profile, and a spline was created from these positions. Perpendicular lines to each pixel of the resting hair bundle position were calculated. For each frame, the intersection of the hair bundle spline and the perpendicular lines were determined, and the measured displacement was calculated as the displacement from the first frame.

**FIGURE 8:**
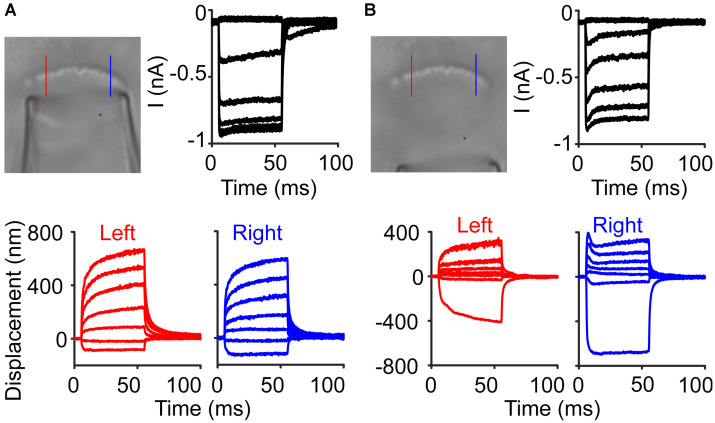
Stimulus artifacts lead to the appearance of current adaptation. **(A)** An inner hair cell was stimulated with a fluid jet to elicit a current response with little adaptation observed in the current. Lines indicate locations where the hair bundle motion was taken in the raw image. **(B)** In the same cell, a larger amount of current adaptation was observed, but the hair bundle motion showed that on the right side of the hair bundle, there was a recoil of the hair bundle, causing an artifactual current decline.

With an incoherent hair bundle, experiments are more susceptible to stimulus artifacts. In one inner hair cell, normal stimulations resulted in MET currents and displacements that we typically observed (Figure 8A). In the same cell, upon moving the stimulation pipette further back, a stimulus artifact appeared due to some cellular debris on one side of the hair bundle and was only visible with slow-motion video (Supplementary Video 2). The stimulus artifact resulted in a slight hair bundle recoil on the right side of the bundle and resulted in what appeared to be a fast time-dependent current decline (Figure 8B). This artifactual motion reinforced the importance of tracking hair bundle motion in every experiment involving the fluid jet to ensure proper stimulation.

### Fluorescent Imaging

Videos of fluorescent dye from fluid jet stimulation, with and without hair cell bundles, using a pipette with a diameter slightly larger than the diameter of the hair bundle, were recorded using a swept-field confocal (Prairie Technologies) on a BX51-WI (Olympus) illuminated with a helium-neon laser at 594 nm. Each pipette was filled with spin-filtered 1 μM Alexa 594 in extracellular solution. Videos were recorded at 1,000 frames per second using a 100x 1.0NA dipping objective (Olympus, Pittsburg, PA, United States).

### Modeling

A MATLAB Simscape model was used to simulate the combination of springs and dashpots. A square force step was used to model the fluid jet step response. The base model used parameters: b_1_ = 8.3 μN/(m/s) (Shepherd and Corey, 1994), b_2_ = 3.4 μN/(m/s), k_1_ = 250 μN/m (Tobin et al., 2019), k_2_ = 2 mN/m, and k_*s*_ = 2 mN/m (Beurg et al., 2008; Tobin et al., 2019). Some parameters were approximated from existing data and others were chosen to generate behavior similar to the hair bundle.

For modeling the fluid flow, we used a 3D computation with the finite element method. The puffer and the tall row of stereocilia in the bundle were configured, as indicated in Figure 3E. Flow through the bundle, which can occur for high amplitude stereocilia splaying, and bundle displacement were not considered. The objective was to determine the pressure and shear stress loading of the stereocilia for different puffer sizes and distances from the bundle. The equations for 3D, incompressible fluid flow (Stokes flow) were the following (Batchelor, 1967):

$$\mu \,\left(u_{,xx}+u_{,yy}+u_{,zz}\right)-p_{,x}=\rho \,u_{,t}$$

$$\mu \,\left(v_{,xx}+v_{,yy}+v_{,zz}\right)-p_{,y}=\rho \,v_{,t}$$

$$\mu \,\left(w_{,xx}+w_{,yy}+w_{,zz}\right)-p_{,z}=\rho \,w_{,t}$$

$$u_{,x}+v_{,y}+w_{,z}=0$$

where *x, y*, and *z* were the Cartesian coordinates, *t* was time, *u, v*, and *w* were the components of velocity in the *x, y*, and *z* directions, *p* was the pressure, *μ* was the viscosity, and *ρ* was the density. Time-dependent solutions are also computed for sinusoidal puffer pressure variation. Because of the small bundle size, the steady-state flow was accurate to well over 10 kHz. These equations were readily solved using the finite element mesh generation and equation solving capability in *Mathematica* (Wolfram, Champaign, IL, United States). The computation required only about 2 min on a laptop (Mac Book Pro, 2.7 GHz). The results were validated by assuring that no significant changes occurred with mesh refinement and that all boundary conditions were satisfied. The differences in the normal and shear stress acting on the front and back surfaces representing the tall row of stereocilia were calculated. This difference multiplied by the distance *z* from the rootlet was then integrated over the height, to obtain the moment acting on each stereocilium about the rootlet. The effective pressure and shear were the values that produce the moment if uniformly distributed.

Parameters for flow calculations: distance puffer face to cilia = 0.47 μm, diameter of puffer = 4.5 and 8 μm, width of cilia row = 5.4 μm, total angle of cilia row = 68.7°, thickness of cilia row = 0.4 μm, length of puffer shown = 4 μm, length of box = 20 μm, height of box = 20 μm (top view), 10 μm (side view), fluid viscosity = 0.7 mPa⋅s, fluid density = 10^3^ kg/m^3^. The pressure on the puffer face was uniform with 1 Pa magnitude, while the pressure at the right end of the box was zero. All the other surfaces were no-slip, i.e., with zero velocity components at each point.

For the hair bundle finite element model, we used the model from Nam et al. (2015). Tip-link stiffness was 4 mN/m, lateral link stiffness was 10 mN/m on axis and 5 mN/m off axis, and the damping coefficient of lateral links was 10 μN/(m/s) on axis and 1 μN/(m/s) off axis. Simulations were run with forces applied to the tallest row of stereocilia with and without lateral links, and displacement of the stereocilia were measured.

### Data Analysis

IX plots used the displacement data from the high-speed imaging taking the displacement values at when the peak current occurred for 50 ms step traces. For multi-pulse protocols, displacement and current were taken 1 ms or 3.9 ms after stimulus onset, because the steps were short (5 ms), and peak currents were not always reached during this time period. Normalized currents (I/I_*max*_) were generated by subtracting leak current, which was defined as the smallest remaining current during the negative steps, and normalizing to the peak current. IX plots were fit with a double Boltzmann equation:

$$y=\frac{I_{m\,a\,x}}{1+e^{Z_{2}\,\left(x_{0}-x\right)}\,\left(1+e^{Z_{1}\,\left(x_{0}-x\right)}\right)}$$

Where Z_1_ and Z_2_ were the slope factors and x_0_ was the set point (Peng et al., 2016; Caprara et al., 2019).

The motion of the hair bundle was also fit with a double exponential equation:

$$y=y_{0}+A_{1}\,e^{-\left(x-x_{0}\right)/\tau_{1}}+A_{2}\,e^{-\left(x-x_{0}\right)/\tau_{2}}$$

**FIGURE 9:**
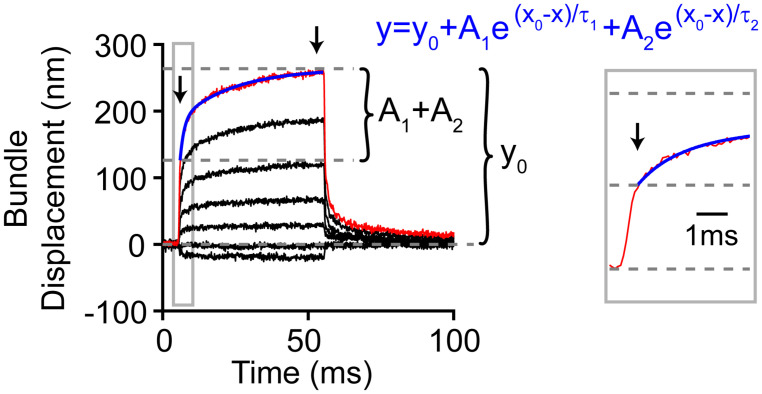
Analysis method for hair bundle creep. Red line highlights the measured motion for the largest step, and blue indicates the double exponential fit (equation in blue) to the creep (gray box zooms in on the onset). Not all the bundle motion was included in the blue fit as shown. The zoom-in view shows the part of the onset that was not included in the fit. During this time, the force stimulus itself was still rising and reached a plateau 0.5 ms after force onset. Thus, the creep kinetics were fit after this. Arrows indicate the time points between which the creep was measured.

Where τ_1_ and τ_2_ were the time constants and A_1_ and A_2_ were the respective amplitudes (Caprara et al., 2019, 2020). Fits were done after the force of the stimulus plateaued, about 0.5 ms after stimulus onset (Caprara et al., 2019; Figure 9). Creep fit parameters were presented as an average of the six largest steps.

In some experiments, the hair bundle’s position in each frame was fit using a quadratic equation:

$$y=a\,x^{2}+b\,x+c$$

Where a was the quadratic coefficient, b was the linear coefficient, and c was a constant. We only used hair bundle positions in the middle parts of the hair bundle approximately from 0.25 to 0.75 of the hair bundle length because the ends of the hair bundle produced artifacts with our position finding algorithm as showcased in Figure 1D with gaps and curling at the hair bundle edges.

All sample sizes (n) were biological replicates of individual cells, where generally only one cell was used per animal. The only exception to this was the fixed tissue experiments, where consecutive cells in the same preparation were used. Each data point was the average of four presentations of the same stimulus intensity (technical replicates), unless otherwise noted.

Data were analyzed using jClamp, MATLAB, and Excel (Microsoft, Redmond, WA, United States). Graphs were created using MATLAB, Origin, and Adobe Illustrator. The mechanosensitive current/maximum mechanosensitive current was used as P_*open*_, where we assumed we observed a P_*open*_ of 100% when current amplitude was maximum. Maximum MET current was the difference between current values elicited from the maximal negative and maximal positive stimulation.

Statistical analysis used Student two-tailed *t*-tests from MATLAB or Excel unless otherwise stated. Paired tests were used when comparing across data points in the same cell and unpaired-unequal variance tests were used to compare data across cell populations. Significance (*p*-values) were as follows, ^∗^*p* < 0.05, ^∗∗^*p* < 0.01, ^∗∗∗^*p* < 0.001, ^****^p < 0.0001. Data were presented as mean ± standard deviation unless otherwise noted. Cohen’s d parameters were calculated using pooled variance.

## Data Availability Statement

The raw data supporting the conclusions of this article will be made available by the authors, without undue reservation.

## Ethics Statement

The animal study was reviewed and approved by the University of Colorado Institutional Animal Care and Use Committee and Stanford University Administrative Panel on Laboratory Animal Care.

## Author Contributions

AP and AR designed the research. AS and AR performed imaging and analyzed the data in Figure 3. DN performed experiments in Figure 2. CS performed the modeling of the 3D fluid flow. GC performed experiments with the flexible fiber in Figures 5, 6. AP performed all other experiments and analysis, developed the analysis code with some contribution from AS, and wrote the manuscript. All authors revised the manuscript.

## Conflict of Interest

The authors declare that the research was conducted in the absence of any commercial or financial relationships that could be construed as a potential conflict of interest.

## Publisher’s Note

All claims expressed in this article are solely those of the authors and do not necessarily represent those of their affiliated organizations, or those of the publisher, the editors and the reviewers. Any product that may be evaluated in this article, or claim that may be made by its manufacturer, is not guaranteed or endorsed by the publisher.

## Supplementary Material

Supplementary Video 1 Video of OHC stimulation from Figure 1. Playback is at 50 fps and of the last largest stimulation.

Supplementary Video 2 Video of non-uniform motion of IHC from Figure 8 showing artifactual relaxation on the right side of the hair bundle. Playback is at 50 fps and of the last largest stimulation.
